# Evaluation of interleukins 8 and 12, CA 15-3 and free circulating DNA as prognostic markers in dogs with mammary tumors

**DOI:** 10.1186/1753-6561-7-S2-O4

**Published:** 2013-04-04

**Authors:** Gabriela B Gelaleti, Camila Leonel, Marina G Moschetta, Bruna V Jardim, Lívia C Ferreira, Juliana R Lopes, Larissa B Maschio, Naiane N Gonçalves, Thaiz F Borin, Gustavo R Martins, Debora APC Zuccari

**Affiliations:** 1 Department of Biology, Graduate Program in Genetics, UNESP/IBILCE, São José do Rio Preto, SP, Brazil; 2 Departament of Molecular Biology, Graduate Program in Health Science, FAMERP, São José do Rio Preto, SP, Brazil; 3 Departament of Molecular Biology, FAMERP, São José do Rio Preto, SP, Brazil

## Background

Mammary tumors of dogs are an excellent model to investigate the clinical and pathological diagnosis and prognosis of cancer. Interleukins play a key role in cancer, particularly interleukin-8, which has tumorigenic and pro-angiogenic properties and interleukin-12, with anti-metastatic and angiogenic properties. The carbohydrate antigen tumor marker (CA 15-3) has important clinical significance in the monitoring of patients with breast cancer and, in addition, free circulating DNA has been considered a candidate biomarker for cancer. The aim of this study was to measure serum levels of interleukin-8, 12, CA 15-3 and to estimate the number of copies of CAN SINE sequences to correlate with clinicopathological parameters and survival.

## Materials and methods

We used enzyme-linked immunosorbent assay and qPCR to evaluate 33 bitches with mammary cancer and 50 control bitches.

## Results

High levels of interleukin-8 were found in bitches with mammary cancer and were correlated with disease progression, lymph node involvement, recurrence and death. Low levels of interleukin-12 were observed in bitches older than 10 years, with longer tumor time course, and, high levels were correlated with high survival rate. High levels of CA 15-3 were correlated with lymph node involvement and death and, in estimating the number of copies of CANSINEs, a significant difference was found regarding the parameters that indicate poor prognosis and low survival.

## Conclusions

Our results show that these proteins and the effect of SINEs can be used as noninvasive prognostic markers in breast cancer research and are useful in predicting progression and tumor recurrence in bitches with mammary cancer.

## Financial support

FAPESP.

